# Effectiveness and Safety of Hybrid Comprehensive Telerehabilitation in Women with Heart Failure—A Subanalysis of the TELEREH-HF Randomized Clinical Trial

**DOI:** 10.3390/jcm15020694

**Published:** 2026-01-15

**Authors:** Ewa Piotrowicz, Renata Główczyńska, Dominika Szalewska, Ilona Kowalik, Piotr Orzechowski, Sławomir Pluta, Zbigniew Kalarus, Anna Mierzyńska, Izabela Jaworska, Robert Irzmański, Ryszard Piotrowicz

**Affiliations:** 1 Telecardiology Center, National Institute of Cardiology, 04-628 Warsaw, Poland; porzechowski@ikard.pl; 2 1st Chair and Department of Cardiology, Medical University of Warsaw, 02-097 Warsaw, Poland; 3 Clinic of Rehabilitation Medicine, Faculty of Health Sciences, Medical University of Gdańsk, 80-210 Gdańsk, Poland; 4 National Institute of Cardiology, 04-628 Warsaw, Polandrpiotrowicz@ikard.pl (R.P.); 5 Department of Cardiology, Congenital Heart Diseases and Electrotherapy, Silesian Center for Heart Diseases, Silesian Medical University, 41-800 Zabrze, Polandi.jaworska@sccs.pl (I.J.); 6 Department of Internal Medicine and Cardiac Rehabilitation, Medical University of Łódź, 90-647 Łódź, Poland

**Keywords:** women, hybrid telerehabilitation, heart failure with reduced ejection fraction

## Abstract

**Background/Objectives**: Despite the known benefits of cardiac rehabilitation, it remains underutilized among women. In particular, little is known about the effectiveness of hybrid comprehensive telerehabilitation (HCTR) in women with heart failure (HF). The purpose of this study was to assess effectiveness and safety of HCTR in women with HF. **Methods**: This analysis formed part of the TELEREH-HF multicenter, randomized trial that enrolled 850 HF patients (NYHA I-III, LVEF ≤ 40%). Patients were randomized 1:1 to HCTR plus usual care (UC) or UC alone. Patients underwent either HCTR (1 week in hospital and 8 weeks at home, five times weekly) or UC with observation. The effectiveness of HCTR was assessed by changes in peak oxygen consumption (VO_2_peak), workload duration (t) in cardiopulmonary exercise test and quality of life (QoL) based on Medical Outcome Survey Short Form 36 Questionnaire (SF-36). Measurements were taken before and after intervention/observation. **Results**: Women constituted 11.5% of the TELEREH-HF study population. Forty women in the HCTR group and 44 women in the UC group completed program and observation, respectively. HCTR resulted in a significant improvement in VO_2_peak (13.4 ± 4.3 vs. 14.3 ± 4.6; 95%CI 0.91 [0.05; 1.77], *p* = 0.038), workload duration (301 ± 162.3 vs. 334 ± 156.6; 95%CI 33 [5; 60], *p* = 0.022) and SF-36 overall score (85.9 ± 13.6 vs. 89.9 ± 13.5; 95%CI 4.0 [0.6; 7.4], *p* = 0.024). These favorable results were not observed in the UC group VO_2_peak (14.2 ± 4.8 vs. 14.2 ± 4.8; 95%CI 0.02 [−1.20; 1.24], *p* = 0.971) and SF-36 overall score (89.1 ± 17.4 vs. 89.5 ± 15.8; 95%CI 4.0 [−2.1; 2.8], *p* = 0.796), except for an increase workload duration (268 ± 138.4 vs. 300 ± 130.1; 95%CI 32 [2; 62], *p* = 0.036). The HCTR group showed a significantly greater improvement in the physical component of QoL than the UC group. In neither group were there deaths nor major adverse events related to exercise training. **Conclusions**: Among women with heart failure, hybrid comprehensive telerehabilitation appears safe and leads to statistically significant although moderate improvements in physical capacity and quality of life. However, due to the small sample size, further studies in larger female populations are needed to confirm these findings.

## 1. Introduction

Heart failure (HF) remains a major public health concern, with significant clinical, psychological, and economic consequences. Women with HF often present with more severe symptoms, lower exercise tolerance, and reduced health-related quality of life (QoL) compared to men, yet they are markedly underrepresented in cardiac rehabilitation programmes [1,2]. Despite strong recommendations in clinical guidelines, cardiac rehabilitation remains underutilised—particularly among female patients—due to factors such as caregiving responsibilities, limited referrals, and restricted access to facility-based services [3,4].

Comprehensive cardiac rehabilitation is a cornerstone of secondary prevention in cardiovascular disease and plays a critical role in improving outcomes in HF [3,4,5,6,7]. Alternative models such as hybrid comprehensive telerehabilitation (HCTR) have been developed to address the barriers limiting participation, especially among women. HCTR is an intervention that combines elements of conventional, in-center cardiac rehabilitation with remote, technology-assisted components. Its concept is based on the integration of two forms of rehabilitation: initially supervised in-center sessions, followed by remotely monitored home-based training sessions during which ECG, target training heart rate, blood pressure, and body mass are monitored. Core components typically include individualized exercise training, educational modules on lifestyle and disease management, regular monitoring of physiological parameters, and structured follow-up care via telecommunication. HCTR differs from conventional cardiac rehabilitation by offering greater flexibility and accessibility through remote components while retaining the benefits of direct professional supervision. Compared with fully remote programs, HCTR retains an in-person component, allowing for hands-on assessment and individualized rehabilitation planning. This model has the potential to increase accessibility, adherence, and long-term engagement in cardiac rehabilitation, particularly among underserved populations [5].

The TELEREH-HF randomised controlled trial demonstrated that HCTR improves functional capacity and QoL in patients with HF with reduced ejection fraction (HFrEF), and that it is safe and feasible for broader clinical implementation [6,8,9]. However, the specific effects of this intervention in women remain unclear due to their low representation in previous studies. Sex-specific evaluations are essential to ensure equitable, evidence-based care. In particular, little is known about the effectiveness of HCTR in women with HF. Therefore, the purpose of this subanalysis of the TELEREH-HF trial was to assess effectiveness and safety of HCTR in women with HF. The primary objective of this subanalysis was to evaluate the impact of HCTR on functional capacity, as measured by peak VO_2_ and exercise duration, and on quality of life, assessed using the SF-36 questionnaire. The secondary objective was to evaluate the safety of the intervention.

## 2. Materials and Methods

The present analysis formed part of TELEREH-HF randomized, multi-center (five centers in Poland), prospective, open-label, parallel-group, controlled study (Clinical Trials.gov NCT 02523560), which enrolled 850 HF patients (New York Heart Association [NYHA] I-III, left ventricular ejection fraction [LVEF] ≤ 40%) who had been hospitalized for worsening HF within six months prior to randomization. Patients were randomized 1:1 to receive HCTR plus usual care (UC) or UC alone. The first patient was recruited on 31 August 2015.

Patients underwent either the HCTR program—which consisted of one week in hospital, followed by eight weeks of home-based training performed five times weekly—or UC with observation. The HCTR intervention was comprehensive and included telecare, tailored home-based telerehabilitation, and remote monitoring of cardiovascular implantable electronic devices. The rehabilitation regimen comprised personalized endurance aerobic training based on Nordic walking, respiratory muscle training, and light resistance and strength exercises. Dietary consultation and interactive nutrition education were conducted in a group format on-site at the center. Additionally, patients were provided with access to the project’s website, where information regarding diet was available. Patients randomized to the UC group were observed and received UC appropriate to their clinical status at their respective centers.

The design, the inclusion and exclusion criteria, the detailed description of the medical team composition, telemonitoring equipment, intervention protocols and primary results of the TELEREH-HF study have been published elsewhere [8,9,10,11,12].

The study adhered to good clinical practice guidelines and was conducted in accordance with the Declaration of Helsinki and applicable Polish regulations. The trial protocol was approved by the local ethics committee (IK-NP-0021-85/1402/13) and all patients provided written informed consent [8,9].

All patients underwent clinical examination, laboratory testing, echocardiography, cardiopulmonary exercise testing (CPET), and psychological assessment both at baseline and after completing the nine-week program.

### 2.1. Cardiopulmonary Exercise Test

The symptom-limited CPET on a treadmill according to a ramp protocol and the ESC guidelines was performed using a Schiller MTM-1500 med; Schiller, Baar, Switzerland [13,14]. Oxygen consumption (VO_2_) was measured continuously using breath-by-breath analysis. The peak VO_2_ value was presented per kilogram of body mass per minute (mL/kg/min). Maximal exercise was defined as the respiratory exchange ratio (RER) ≥ 1.

### 2.2. Health-Related Quality of Life Assessment

The Medical Outcome Survey Short Form 36 Questionnaire (SF-36) was used to assess QoL. The SF-36 consists of two major domains (physical and mental QoL) and various subscales [15]. Higher scores indicate a better QoL. We chose the SF-36 because this questionnaire provides detailed information on both physical and mental health domains, which was particularly relevant for our study assessing the broader impact of HCTR.

The effectiveness of HCTR was assessed by changes delta (Δ) in peak VO_2_, workload duration (t) in CPET and QoL based on SF-36. Measurements were made before and after intervention/observation.

### 2.3. Statistical Analyses

Numerical variables are presented as means and SD or median and interquartile ranges (IQR), as appropriate. The distribution of data was examined by the Shapiro–Wilks test. Depending on the distribution comparisons between groups were made using Student’s *t*-test or the Mann–Whitney test. For comparisons within groups, appropriate paired tests were used: paired Student’s *t*-test or the Wilcoxon signed-rank test. Categorical variables are shown as counts and percentages. The χ^2^-test or Fisher’s exact test was performed to verify equality of proportions. 95% confidence intervals (Cis) were calculated for changes in VO_2_, workload duration, and quality of life. Cohen’s d was used as the measure of effect size, with 0.2 considered a weak effect, 0.5 a moderate effect, and 0.8 a strong effect. Due to the small size of the subgroups (this analysis represents a sub-study), complex analysis designs were not used, and no correction for multiple comparisons was made. All tests were two-sided, and a *p*-value < 0.05 was considered statistically significant. All analyses were conducted using SAS software version 9.4.

## 3. Results

Of the 850 patients randomized to TELEREH-HF trial, 425 were assigned to the HCTR group and 425 to the UC group. Women constituted 11.5% of study population (48 in the HCTR group and 49 in the UC group). There were 2 women among the 27 patients who did not undergo telerehabilitation. One woman was unable to participate due to technical difficulties operating the telerehabilitation equipment, and the other due to new-onset comorbidities.

**Figure 1:**
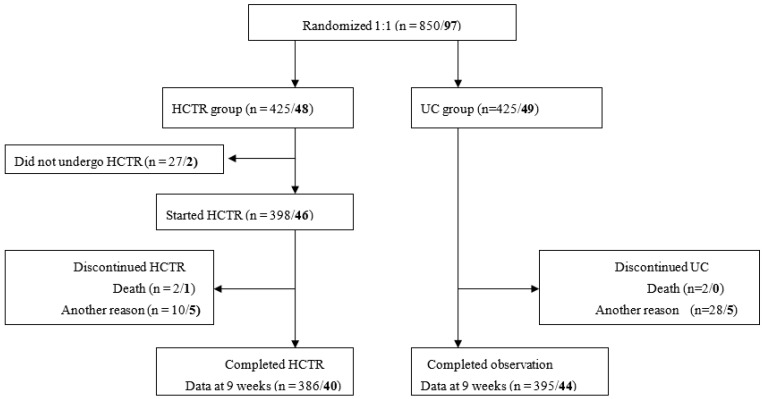
Flow of the entire patient population/women through the TELERH-HF study. HCTR—hybrid comprehensive telerehabilitation, UC—usual care.

Among the 46 women who started telerehabilitation, five discontinued HCTR due to returning to work, and one died following postoperative complications (unrelated to the intervention). This subanalysis ultimately included 40 women in the HCTR group who completed the 9-week hybrid telerehabilitation program and 44 women in the UC group who completed the observation period Figure 1. Adherence to the 9-week HCTR program was high—approximately 89%.

The baseline characteristics of women in both groups (HCTR and UC) enrolled in the TELEREH-HF trial are presented in Table 1. Neither sodium–glucose cotransporter-2 (SGLT2) inhibitors nor glucagon-like peptide-1 (GLP-1) receptor agonists were used in the study population. The randomization and intervention/observation period took place before these drug classes became part of guideline-recommended therapy for HF; therefore, none of the participants received these medications. No significant differences were observed between groups at baseline in demographic data, clinical parameters, or treatment. The mean age of women was relatively low in both groups (58.8 years in HCTR; 63.0 years in UC; *p* = 0.089) and LVEF was 31.9% and 33.8% in the HCTR and UC groups, respectively (*p* = 0.153). Ischemic etiology of HF was diagnosed with comparable frequency: 40% in the HCTR group vs. 47.7% in the UC group (*p* = 0.476).

Cardiopulmonary exercise test results and QoL scores at baseline and after the 9-week intervention/observation period are presented in Table 2 and Table 3. At baseline the study groups did not differ significantly in workload duration and peak VO_2_. In both groups workload duration improved significantly compared to baseline (*p* = 0.022 in HCTR and *p* = 0.036 in UC, respectively). HCTR also resulted in a significant improvement in peak VO_2_ (*p* = 0.038), whereas no significant change in this parameter was observed in the UC group; so that in the HCTR group the increase in peak VO_2_ after 9 weeks occurred in 29 patients (72.5%), while in the UC group only in 19 patients (46.3%), *p* = 0.017.

Following the intervention, the HCTR group showed a significant improvement in overall QoL compared to baseline (∆ = 4.0 [0.6; 7.4]; *p* = 0.024) and in the physical domain of QoL (∆ = 2.0 [0.05; 3.9]; *p* = 0.044). No significant changes were observed in the mental domain of QoL in the HCTR group. However, no statistically significant differences were found in overall QoL or its domains in the UC group after the observation period.

In the baseline assessment, the HCTR group included 35% of patients who were overweight and 30% who were obese; in the UC group, these proportions were 36.4% and 34.1%, respectively (Table 1). Among patients with overweight or obesity, body mass did not change significantly after the intervention/observation period in either group. In the HCTR group, median (Q1–Q3) body mass was 73 (64–84) kg at baseline and 74 (64–84) kg after 9 weeks (*p* = 0.864). In the UC group, the corresponding values were 74 (65–85) kg at baseline and 75 (64.5–85.5) kg after 9 weeks (*p* = 0.811).

No deaths or major adverse events related to exercise training occurred in either group.

## 4. Discussion

Cardiac rehabilitation is a well-established intervention proven to reduce recurrent cardiac events, enhance functional capacity, and improve QoL. In our study, we observed a significantly greater improvement in the physical component of QoL in the HCTR group compared with UC. The HCTR group achieved a significant increase in peak VO_2_, demonstrating a clear enhancement in exercise capacity among women. Moreover, a substantially higher proportion of women in the HCTR group improved their peak VO_2_ compared with those receiving UC, highlighting the superior functional benefits of the hybrid telerehabilitation approach. We found significant improvements in overall QoL and its physical domain in the HCTR group, whereas no such improvements were observed in the UC group; QoL in the mental domain remained unchanged in both groups. Importantly, in our study, the hybrid telerehabilitation program demonstrated an excellent safety profile, with no deaths or major exercise-related adverse events.

Although historically underrecognized, HF in women presents distinct epidemiological, pathophysiological, and clinical features compared to men. Women with HF often exhibit different symptoms, respond differently to treatments, and face unique psychosocial and healthcare access challenges. Understanding these differences is essential for developing effective, gender-sensitive diagnostic and therapeutic strategies.

Women develop HF later in life than men and more frequently exhibit non-ischemic etiologies such as hypertensive heart disease, valvular dysfunction, and HF with preserved ejection fraction (HFpEF). These phenotypes are often characterized by distinct symptom profiles, including greater dyspnea, fatigue, and exercise intolerance, which can complicate diagnosis and management.

Women with HF often report lower QoL and higher levels of depression and anxiety than their male counterparts. These psychosocial challenges can negatively impact self-care behaviors and adherence to treatment. Social support networks, counseling, and mental health care are therefore essential components of comprehensive HF management in women.

Given these differences, several mechanisms may underlie sex-specific responses to telerehabilitation. Biologically, HF phenotypes predominant in women—such as HFpEF and microvascular dysfunction—are marked by exercise intolerance and impaired peripheral oxygen extraction, which may respond particularly well to the progressive aerobic conditioning central to telerehabilitation. Sex-related differences in autonomic regulation, resting heart rate, heart rate variability, and thoracic anatomy may also influence how physiological signals are captured and interpreted by remote monitoring systems, potentially affecting the personalization of exercise loads. Psychosocial factors further shape women’s engagement: higher levels of fatigue, anxiety, and depressive symptoms can hinder participation in traditional center-based programs, whereas the privacy and flexibility of home-based training may enhance emotional safety and comfort. In addition, caregiving duties and time constraints—a well-recognized burden for many women—are alleviated by the reduced travel and scheduling demands of telerehabilitation. Adherence-related mechanisms are also relevant. Though older women may initially express lower confidence with digital tools, structured education, intuitive interfaces, and regular feedback have been shown to improve digital competence and sustain engagement. Women often value frequent reassurance and personalized communication, and telehealth platforms that provide continuous feedback and support may strengthen motivation and adherence.

Extensive research consistently shows that cardiac rehabilitation reduces mortality, improves physical fitness, and enhances psychological well-being in both sexes [16]. For women specifically, it offers an opportunity to address modifiable risk factors such as hypertension, hyperlipidemia, diabetes, obesity, and depression—which are highly prevalent in female cardiovascular populations. Additionally, women more commonly present with non-obstructive coronary artery disease, microvascular dysfunction, and atypical symptoms, which may complicate diagnosis and management [17]. Cardiac rehabilitation provides a personalized and holistic framework well suited to addressing these gender-specific clinical nuances.

Although cardiac rehabilitation is highly effective, women consistently participate less often than men. Referral biases, competing caregiving responsibilities, socioeconomic constraints, and psychological barriers, such as lower self-efficacy and fear of exercise contribute to this gap. Despite its benefits, traditional center-based cardiac rehabilitation faces notable challenges, including limited accessibility, low participation rates, and logistical difficulties-issues that disproportionately affect women. In our TELEREH-HF study, women represented only 11.5% of the population, with similar proportions in both the HCTR and UC groups, reflecting the well-documented underrepresentation of women in rehabilitation programs. Underrepresentation limits evidence-based, gender-informed decision-making and may contribute to poorer outcomes after cardiovascular events.

Several factors contribute to this disparity. Physicians may be less likely to refer women, particularly younger women or those presenting with atypical symptoms, to rehabilitation programs [4]. Sociocultural responsibilities, including caregiving and household duties, often restrict women’s ability to attend scheduled sessions [18]. Additionally, higher rates of depression and anxiety, lower self-efficacy, and feelings of vulnerability can hinder women’s participation and adherence [19]. Although cardiovascular disease remains the leading cause of death among women worldwide, they continue to be underreferred to and underrepresented in cardiac rehabilitation, frequently experiencing poorer post-event outcomes than men.

Cardiac rehabilitation, including structured exercise training, education, and counseling is a proven strategy for reducing mortality and improving QoL [20]. To maximize benefit for women, however, programs must address gender-specific physiological and psychosocial barriers. Understanding and reducing these obstacles is essential for improving rehabilitation engagement and outcomes among women.

Telerehabilitation, the remote delivery of cardiac rehabilitation through digital and telecommunication technologies, presents a promising and increasingly validated alternative. Remote monitoring and hybrid cardiac telerehabilitation offer promising strategies to reduce disparities, yet sex-specific performance data remain scarce. Physiological parameters commonly used in remote monitoring, such as heart rate variability, thoracic impedance, arrhythmia burden, and physical activity levels may differ between men and women. These variations could influence the accuracy and predictive value of telerehabilitation algorithms, which were predominantly validated in male populations [21].

Multiple studies have shown telerehabilitation to be comparable in effectiveness to traditional, center-based rehabilitation. A meta-analysis by Thomas et al. [22] demonstrated that digital home-based programs result in similar improvements in peak oxygen uptake, exercise capacity, and health-related QoL. In the TELEREH-HF study, we further confirmed that telerehabilitation can enhance physical capacity in women with HF, evidenced by increased peak VO_2_ and prolonged workload duration.

Recent evidence also supports the broader effectiveness of telerehabilitation. A review by Jin et al. [23] reported that programs incorporating structured exercise, education, and behavioral counseling significantly improve physical activity levels and patient adherence compared to standard care. These findings reinforce the feasibility and clinical value of telerehabilitation across diverse patient groups.

Beyond clinical efficacy, telerehabilitation offers several practical advantages. It addresses geographic and transportation barriers, making it particularly suitable for individuals living in rural or underserved regions [24]. It also reduces travel-related burden, lowers facility and system costs, and has the potential to decrease hospital readmissions.

Flexible scheduling, reduced travel burden, and the ability to exercise in a home environment may improve participation and adherence. Findings from the TELEREH-HF study support these advantages: women in the HCTR group demonstrated significant improvement in exercise capacity, including increased peak VO_2_ and prolonged workload duration. A greater proportion of women improved peak VO_2_ compared with usual care, underscoring the superior functional benefit of HCTR. QoL also improved significantly, particularly in the physical domain, whereas no meaningful changes were observed among women receiving usual care.

Given the strong potential of telerehabilitation to reduce access barriers and enhance engagement, future research must incorporate balanced male–female representation and report sex-disaggregated outcomes. Such efforts are essential for ensuring that digital innovations in HF management benefit women equitably and improve long-term clinical trajectories.

Limitation. This study has several limitations that should be acknowledged. First, the sample size of female participants was relatively small, reflecting the overall underrepresentation of women in the TELEREH-HF trial. As a result, the statistical power to detect differences between groups may be limited, and findings should be interpreted with caution. Second, the study population consisted solely of patients from centers in Poland, which may limit the generalizability of the results to more diverse populations or healthcare settings. Third, psychological and social factors could influence participation and outcomes—such as motivation, support systems, or socioeconomic status—were not extensively assessed. Finally, the follow-up period was limited to nine weeks; therefore, the long-term effects of HCTR on clinical outcomes and QoL in women with HF remain unknown and warrant further investigation. Furthermore, this subanalysis was conducted post hoc, which limits the interpretability of the results. The study was open-label by design; therefore, a limitation is the lack of blinding of outcome assessors, which would have reduced potential bias.

## 5. Conclusions

Among women with heart failure, hybrid comprehensive telerehabilitation appears safe and leads to statistically significant although moderate improvements in physical capacity and quality of life. However, due to the small sample size, further studies in larger female populations are needed to confirm these findings.

## Figures and Tables

**Table 1:** Baseline characteristics.

	HCTR Group (*n* = 40)	UC Group (*n* = 44)	*p*
Age (years). mean ± SD	58.8 ± 13.3	63.0 ± 8.3	0.089
Left Ventricular Ejection Fraction (%). mean ± SD	31.9 ± 6.6	33.8 ± 5.5	0.153
Body Mass Index (kg/m^2^)	27.8 ± 5.9	28.6 ± 5.4	0.523
Body Mass Index (kg/m^2^) ≤ 25.0	14 (35.0)	13 (29.5)	0.855
Body Mass Index (kg/m^2^) > 25.0 < 30.0	14 (35.0)	16 (36.4)	
Body Mass Index (kg/m^2^) ≥ 30.0	12 (30.0)	15 (34.1)	
Atrial fibrillation or atrial flutter. *n* (%)	5 (12.5%)	5 (11.4)	1.00
Etiology of heart failure. *n* (%)			
Ischaemic	16 (40.0)	21 (47.7)	0.476
Non-ischaemic	24 (60.0)	23 (52.3)	
Coronary artery disease	16 (40.0)	20 (45.4)	0.614
Myocardial infarction	15 (37.5)	19 (43.2)	0.596
Angioplasty	13 (32.5)	15 (34.1)	0.877
Coronary artery bypass grafting	3 (7.5)	3 (6.8)	1.00
Hypertension	22 (55.0)	25 (56.8)	0.867
Diabetes	9 (22.5)	12 (27.3)	0.614
Stroke	2 (5.0)	3 (6.8)	1.00
Chronic kidney disease	10 (25.0)	8 (18.2)	0.447
Hyperlipidemia	18 (45.0)	18 (40.9)	0.705
NYHA I. *n* (%)	2 (5.0)	2 (4.6)	0.845
NYHA II. *n* (%)	31 (77.5)	32 (72.7)	
NYHA III. *n* (%)	7 (17.5)	10 (22.7)	
Beta-blocker	38 (95.0)	42 (95.3)	1.00
ACEI/ARB	36 (90.0)	38 (86.4)	0.741
Digoxin	3 (7.5)	7 (15.9)	0.319
Loop diuretics	32 (80.0)	31 (70.4)	0.313
Spironolactone/eplerenone	33 (82.5)	31 (70.4)	0.195
Aspirin/clopidogrel	18 (45.0)	24 (54.5)	0.382
Anticoagulants	10 (25.0)	10 (22.7)	0.807
Statins	29 (72.5)	30 (68.2)	0.666
CIEDs	29 (72.5)	28 (63.6)	0.385
Implantable cardioverter–defibrillator	15 (51.7)	14 (50.0)	1.00
CRT-P	1 (3.4)	0 (0)	
CRT-D	12 (41.4)	13 (46.3)	
PM	1 (3.5)	1 (3.6)	

Abbreviations: NYHA—New York Heart Association class; ACEI—angiotensin-converting enzyme inhibitors; ARB—angiotensin receptor blockers; CIEDs—cardiovascular implantable electronic devices; CRT-P—cardiac resynchronization therapy; CRT-D—cardiac resynchronization therapy and cardioverter–defibrillator; PM—pacemaker.

**Table 2:** Results—effectiveness of hybrid comprehensive telerehabilitation in women with heart failure.

Parameters	Hybrid Comprehensive Telerehabilitation (HCTR) Group				Usual Care (UC) Group			
	Baseline	9th Week	Δ [95% CI]	*p*	Baseline	9th Week	Δ [95% CI]	*p*
Workload duration (s)	301 ± 162.3	334 ± 156.6	33 [5; 60]	0.022	268 ± 138.4	300 ± 130.1	32 [2; 62]	0.036
peakVO_2_ (mL/kg/min)	13.4 ± 4.7	14.3 ± 4.6	0.91 [0.05; 1.77]	0.038	14.2 ± 4.8	14.2 ± 4.8	0.02 [−1.20; 1.24]	0.971
SF36 Physical Component Score	39.8 ± 6.5	41.8 ± 6.7	2.0 [0.05; 3.9]	0.044	41.1 ± 8.7	40.4 ± 7.1	−0.7 [−2.1; 0.5]	0.239
SF36 Mental Component Score	46.1 ± 11.1	48.1± 10.1	2.0 [−0.5; 4.6]	0.118	48.0 ± 12.7	49.1 ± 11.5	1.1 [−1.2; 3.4]	0.338
SF 36—overall score	85.9 ± 13.6	89.9 ± 13.5	4.0 [0.6; 7.4]	0.024	89.1 ± 17.4	89.5 ± 15.8	0.4 [−2.1; 2.8]	0.796

SF-36—The Medical Outcome Survey Short Form 36 Questionnaire.

**Table 3:** Results—Cohen’s d; effectiveness of hybrid comprehensive telerehabilitation in women with heart failure.

	Cohen’s d	
Parameters	Hybrid Comprehensive Telerehabilitation (HCTR) Group	Usual Care (UC) Group
Workload duration (s)	0.38	0.33
peakVO_2_ (mL/kg/min)	0.34	0.01
SF36 Physical Component Score	0.33	0.16
SF36 Mental Component Score	0.25	0.15
SF 36—overall score	0.37	0.05

SF-36—The Medical Outcome Survey Short Form 36 Questionnaire.

## Data Availability

The data used to support the findings of this study are included within the article.

## Abbreviations

The following abbreviations are used in this manuscript:

HF	Heart Failure
QoL	Quality of life
HCTR	Hybrid Comprehensive TeleRehabilitation
LVEF	Left Ventricular Ejection Fraction
UC	Usual Care
CPET	CardioPulmonary Exercise Test
NYHA	New York Heart Association
SF-36	Medical Outcome Survey Short Form 36 Questionnaire
